# An efficient rAAV vector for protein expression in cortical parvalbumin expressing interneurons

**DOI:** 10.1038/s41598-022-21867-0

**Published:** 2022-10-25

**Authors:** Tatiana Tkatch, Kristina Rysevaite-Kyguoliene, Ignas Sabeckis, Deimante Sabeckiene, Dainius H. Pauza, Gytis Baranauskas

**Affiliations:** 1 Neurophysiology Laboratory, Neuroscience Institute, Lithuanian University of Health Sciences, Kaunas, Lithuania; 2 Anatomy Institute, Lithuanian University of Health Sciences, Kaunas, Lithuania; 3 Present Address: Department of Physiology, Northwestern University, Chicago, IL USA

**Keywords:** Transcriptional regulatory elements, Genetic transduction, Genetic vectors, Optogenetics

## Abstract

Recombinant adeno—associated viruses (rAAV) are extensively used in both research and clinical applications. Despite significant advances, there is a lack of short promoters able to drive the expression of virus delivered genes in specific classes of neurons. We designed an efficient rAAV vector suitable for the rAAV-mediated gene expression in cortical interneurons, mainly in the parvalbumin expressing cells. The vector includes a short parvalbumin promoter and a specialized poly(A) sequence. The degree of conservation of the parvalbumin gene adjoining non-coding regions was used in both the promoter design and the selection of the poly(A) sequence. The specificity was established by co-localizing the fluorescence of the virus delivered eGFP and the antibody for a neuronal marker. rAAV particles were injected in the visual cortex area V1/V2 of adult rats (2–4 months old). Neurons expressing the virus delivered eGFP were mainly positive for interneuronal markers: 66.5 ± 2.8% for parvalbumin, 14.6 ± 2.4% for somatostatin, 7.1 ± 1.2% for vasoactive intestinal peptide, 2.8 ± 0.6% for cholecystokinin. Meanwhile, only 2.1 ± 0.5% were positive for CaMKII, a marker for principal cells in the cortex. The efficiency of the construct was verified by optogenetic experiments: the expression of the virus delivered ChR2 channels was sufficient to evoke by blue light laser high frequency bursts of action potentials in putative fast spiking neurons. We conclude that our promoter allows highly specific expression of the rAAV delivered cDNAs in cortical interneurons with a strong preference for the parvalbumin positive cells.

## Introduction

rAAVs are widely used in research as a tool for targeted protein expression, mainly in non-dividing cells such as neurons ^1–3^. In fact, rAAVs are the main gene delivery vehicles in optogenetic experiments that are reshaping many areas of neuroscience ^1^. There are several reasons for such a success story of rAAV use: these viruses are non-pathogenic, they do not divide, and, consequently, pose minimal risks during their handling ^1,4^. In addition, under favourable conditions most cells can be infected with rAAVs and express viral proteins ^5^. Because of very low pathogenic risks, rAAVs are the main tool used in gene therapy clinical trials with several treatments approved both in Europe and USA ^3^. Nevertheless, one of the major limiting factors in rAAV use is their low packaging capacity: at most 4–5 kb sequence can be inserted into the virus genome ^1,6^. Although there is some progress in the attempts to overcome this limitation ^7^, it remains a major challenge when it is required to achieve a cell-type specific expression of rAAV delivered genes. Cell-type specific protein expression is affected by genomic sequences that can be several thousands of the DNA base pairs away from the protein encoding sequence ^8,9^. Often, there is no detailed knowledge which parts of these long sequence are crucial to achieve high protein expression specificity. Because of low capacity of rAVVs, it is not possible to use the whole intergenomic sequence and a more detailed knowledge is required. During the last ~ 5 years a rapid progress was made in search for such short promoters ^10–14^. For example, it was demonstrated that the mDlx enhancer can achieve > 90% specificity for interneurons without a clear preference for a single type ^11^. A massive screen of enhancers, one type of gene expression regulators ^9^, enabled to determine several additional short sequences that were shown to allow a highly specific expression of rAAV proteins in a single class of interneurons ^12,14^. For instance, E2 and E29 sequences, derived from intronic and intergenic regions near the transcriptional start site of Scn1a and Inpp5j respectively, enable rAAV virus protein expression in interneurons positive for a Ca^2+^ binding protein parvalbumin (PV) with a > 90% specificity ^14^. These interneurons are of high interest both for basic research and clinicians. In the cortex PV neurons are almost exclusively GABAergic, thus, inhibitory, and most of them can sustain very high frequency firing, > 150 Hz ^15,16^. Therefore, they can control the overall cortical activity ^15^. These neurons have been implicated in several diseases such as epilepsy and schizophrenia ^17–19^. Because of such strong need, we believe that the availability of several promoters with the same or similar level of specificity may lead to further improvements of our understanding about regulation of gene expression and the development of even more specific promoters. For instance, it was shown that with a combination of two viruses a much higher degree of specificity can be achieved than with a single rAAV vector^20^. In addition, there is still relative paucity of information on the specificity of particular regulatory sequences. Therefore, here we present data on a promoter and poly(A) sequence designed by our group that is highly specific for cortical interneurons with strong preference for PV expressing cells.

## Methods

### In silico PV promoter analysis

To find putative PV enhancer-promoter sequences conserved among several mammalian species we aligned 15 kb upstream sequences: *Rattus norvegicus* chromosome 7 whole genome shotgun sequence (CM000237.2) region complement (106125600–106140601), *Mus musculus* genomic scaffold (CH466545.1) region (28380542–28395543) complement, *Homo sapiens* genomic scaffold (CH471095.1) region (15277176–15292177) complement. We used NCBI Blastn (somewhat similar sequences) program to align rat and human sequences and then checked if they are among megablast (highly similar sequences) alignment when rat and mouse sequences were used. Only the sequences confirmed by such megablast screen were used for construction of the promoter. Thus, the megablast sequence similarity algorithm was used for homology cutoffs.

We found 5 sequence blocs (5′ untranslated region of PV mRNA was included) with high homology among these species.

**Figure 1:**
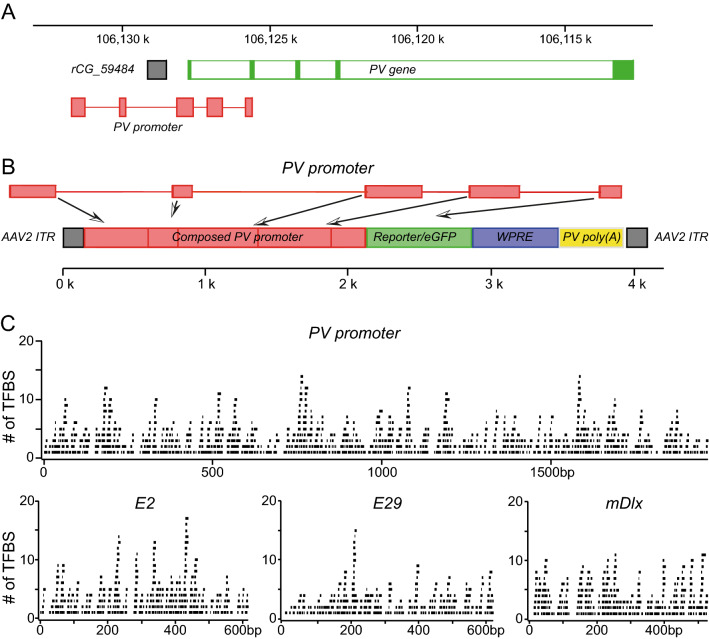
Schematic representation of the promoter design. (**A**) A section of *Rattus norvegicus* chromosome 7 whole genome shotgun sequence (CM000237.2) is shown that includes PV gene (green filled bars represent exons while open bars represent introns) and a ~ 10 kb upstream sequence. Boxes in light red indicate sequence regions used for the promoter construction. (**B**) The rAAV2 construct used in this study. The actual length of the PV promoter shown above is slightly out of scale. WPRE stands for the Woodchuck Hepatitis Virus Posttranscriptional Regulatory Element and ITR for the inverted terminal repeat. (**C**) Transcription factor binding site (TFBS) analysis for PV promoter and three enhancers that were used for selective expression of proteins in cortical interneurons. Each panel shows the promoter or an enhancer sequence with black bars above indicating the predicted TFBS, a single bar corresponds to one TFBS. For details see methods.

To build transcription factor binding site diagrams (Fig. 1C) we used a PROMO for such binding side prediction ^21,22^. PROMO uses TRANSFAC database for predictions. To obtain the conserved binding sites only we used only human transcriptional factors on the rat or mouse sequence as it was done previously in a similar analysis for E2 and E29 enhancers ^14^.

### Cloning and rAAV production

Large rat genomic DNA fragments containing these aforementioned blocks were amplified using Platinum Taq High Fidelity DNA Polymerase (Thermo Fisher) and then the purified PCR fragments were cloned into pcDNA3.1 plasmid (Invitrogen). From these plasmids smaller fragments were amplified and put together. *Block 1* corresponds to complement sequence 106131296–106131746 (CM000237.2), *block 2* corresponds to complement sequence 106129909–106130120, *block 3* corresponds to complement sequence 106127620–106128180, block 4 corresponds to complement sequence 106126631–106127143, *block 4* corresponds to complement sequence 106125613–106125846.

In the following, the primers used for fragment cloning are listed.

- Fragment 1 (*block 1*):
  - Upper primer TAAGCCAAGCTTGCCTTGAGGGTCCTC.
  - Lower primer ACACTGCAAGCTTAGAGGGTGAGGTGT.
- Fragment 2 (*block 2* and *block 3*):
  - Upper primer ACACCTCACCCTCTAAGCTTGCAGTGT.
  - Lower primer CCACAGCCTCCATATTGATTCTAGAG.
- Fragment 3 (*block 4*):
  - Upper primer GGATAGCCATGCCGCTATTTCTCTAGA.
  - Lower primer AGATCATACTTTCTTTCTAGACTACCAGGT.
- Fragment 4 (*block 5*):
  - Upper primer TGGTAGTCTAGAAAGTATGATCTGATG.
  - Lower primer CATCGGGATCCTGCAACTGTTTAAGCG.

Primers for final cloning were as follows (restriction enzymes used in the final cloning are indicated in the brackets):

- Fragment1 (MluI/BsrGI):
  - Upper primer TAAGACGCGTACGGGGAGAATCAGCTCAG,
  - Lower primer TAGCTGTACACTCATACATACATTCCCCA.
- Fragment2 (BsrGI/KpnI):
  - Upper primer AGCATGTACAAGACTGACTAGGTTAATC.
  - Lower primer CATGGGTACCTACTGCTCTTGTGATCTAG.
- Fragment3 (KpnI/XbaI):
  - Upper primer TATAGGTACCCCCACCCCACAGTGTCT.
  - Lower primer GAGCTCTAGAGAAATAGCGGCATGGCTA.
- Fragment4 (XbaI/BamHI):
  - Upper primer AGCTTCTAGAGACTCCAGGCTCACTATG.
  - Lower primer TATAGGATCCTGTGTGGGACCTGAGG.
- Fragment5 (BamHI/AgeI):
  - Upper primer ATATGGATCCTGATCAAGCCCCCTGAAT.
  - Lower primer TTAAACCGGTTTAAGCGGGCAGAGCAA.

Finally, the combined 1.97 kb fragment was cloned into pAAV-hSyn-eGFP (a gift from Bryan Roth, Addgene plasmid # 50465) instead of hSyn promoter.

3′untranslated region of PV mRNA showed high homology also, so we substituted hGH poly(A) signal for native PV 3′untranslated region amplified using following primers:

- Upper primer GATCCTCGAGTGGCCGAAAGCTAAGTGG.
- Lower primer TAATCTCGAGCCTGGGTCCTCCCTACA.

Viral particles of serotype 2 with a wild type of capsid were purchased from Creative Biogene (Shirley, NY 11967, USA), the stated concentration was 10^13^ vg/mL.

The PV_CHR134_eYFP virus used in optogenetic experiments was similar to the first virus except for the eGFP part that was substituted with a modified channelrhodopsin channel ChR2 with a point mutation H134R and a covalently attached eYFP probe^23^. The AVV5 viral particles were purchased from Virovek (Hayward, CA, USA), the stated concentration was 2 × 10^13^ vg/mL.

### Viral particle in vivo injection

All procedures were carried out in accordance with the European Communities Council Directive of 22 September 2010 on the protection of animals used for scientific purposes (2010/63/EEC) and were approved by the Animal Care and Use Committee of the State Food and Veterinary Service of Lithuania (No. G2-147 of May 13, 2020). All methods involving animals were carried on in accordance with ARRIVE guidelines^24–26^. In this type of research, there is no control group of animals because we compare co-expression of virus delivered and marker proteins in neurons of the same animal. Neurons expressing virus delivered eGFP represent a control group in such experiments. For all major findings, at least 3 animals were used.

Standard procedures were used for virus injections. Briefly, 18 Wistar rats of both sexes, weighting 180–450 g (1.5–4 months old), were anesthetized with a mix of xylazine chloride (Eurovet Animal Health B.V., Bladel, Netherlands, 10 mg/kg), ketamine (Richter Pharma AG, Wels, Austria, 60 mg/kg) and butorphanol (Richter Pharma AG, Wels, Austria, 0.4 mg/kg), delivered intraperitoneally. The depth of anesthesia was monitored by testing for the absence of hind limb withdrawal reflex following a pinch of the paw. To maintain the depth of anaesthesia, additional doses of the mix were used as needed for the duration of the experiment. Under anaesthesia, the body temperature was maintained at 36–38 °C with a heating pad. The anesthetized animal was placed in a stereotaxic apparatus (World Precision Instruments, Sarasota, FL, USA). A small craniotomy (approximately 1 × 1 mm) was made in the parietal bone right above the visual cortex areas V1/V2 (the injection coordinates were 1–2 mm rostral to the lambda and 2–3 mm lateral to the midline). To facilitate glass pipette penetration, a small cut of the dura mater was made. Before injection, virus particles (10^13^ particles/mL) were mixed with a warmed phosphate buffer solution (ratio 1:1) and 0.1–0. 5 µL of the mix was used for injection with a pipette made from a borosilicate glass capillary, corresponding to the total number of injected viral particles of 0.5–2.5 × 10^9^. The injection depth was between 700 and 1200 µm.

Following injection, rats were returned to home cage. Care was taken to avoid any infections. The analysis of brain slices was performed 10 to 28 days post-injection. Time post-injection affects the expression level of transduced cells and after initial increase usually plateaus after 4–6 weeks^1^. However, to the best of our knowledge time post-injection does not affect the specificity of transgene expression and we did not find any correlation between post-injection time and the fraction of co-labelled neurons.

Similarly, no correlation between the age and the sex of the rats and the fraction of an antibody labelled eGFP neurons was found. Therefore, data from all rats of different ages and sexes were pooled. The breakdown of the sexes, ages, and weights of the rats used per each injection is provided in the “Supplementary information”.

#### Tissue sectioning

Thoracotomy was performed and then an incision was made in the right atrium followed by an ample perfusion of the hearts in situ with a phosphate-buffered saline (PBS) via a syringe needle inserted into the left ventricle^27^. Blood wash out from the vessels and the heart was controlled visually. Then, the prefixation with 4% paraformaldehyde (PFA) solution in PBS was applied. The composition of PBS was (in mM): 137 NaCl; 2,7 KCl; 10 Na_2_HPO_4_; 2 KH_2_PO_4_. Brain was exposed and extirpated under a dissecting microscope Stemi 2000 (Carl Zeiss, Jena, Germany). The extracted whole brain was sliced at a coronal plane using Acrylic Adult Rat Brain Slicer (Vector laboratories, Burlingame, CA, USA) and the taken several thick (~ 2 mm) slices at the level of occipital lobes were fixed for 3 h in 4% PFA in PBS. Then those slices were washed 3 × 10 min in PBS, cryoprotected by immersion in PBS containing 30% sucrose and 0.05% sodium azide at 4 °C for 24 h and frozen embedded in tissue-freezing medium (Triangle Biomedical Sciences, USA) using a liquid nitrogen. Fifty-micron serial sections of the SC were prepared from the aforementioned slices employing a cryomicrotome CryoStar NX70 (Thermo Fisher Scientific, USA). The sections were immersed into PBS containing 0.05% sodium azide and left at 4 °C until immunohistochemical reactions in free floating sections were performed.

#### Immunohistochemistry

Prior to incubation with antibodies, all SC sections were left for 50 min in solution containing 1 part DMSO and 10 parts PBS with 1% Triton X-100. Next, sections were rinsed three times for 10 min in pure PBS solution. Nonspecific binding sites in the tissue were blocked by incubation in 5% normal donkey serum in PBS for 1 h. After washing in PBS 3 × 10 min., the sections were soaked in microchambers with a solution of primary antibody (Table 1). Sections were left in a refrigerator at 4 °C overnight. Then sections were rinsed in PBS 3 × 10 min and secondary antibodies (Table 1) were applied at least for 2 h. Finally, specimens were again rinsed in PBS 3 × 10 min. Both the primary and the secondary antibodies were diluted in the Antibody Diluent ab64211 (Abcam, Cambridge, UK). Afterwards, the free-floating SC sections were carefully transferred on the microscope slides. All preparations were cover-slipped using mounting medium (Vectashield, Vector Laboratories, USA) and sealed with clear nail polish.

**Table 1:** Primary and secondary antibodies used in the study.

Antigen	Host	Dilution	Catalogue code	Supplier
**Primary**				
CAMKII	Mouse	1:500	MA1-048	Invitrogen^a^
Cholecystokinin 8	Mouse	1:500	ab37274	Abcam^b^
Somatostatin	Rabbit	1:500	PA5-82678	Invitrogen^a^
Parvalbumin	Rabbit	1:500	ab11427	Abcam^b^
VIP	Mouse	1:500	ab30680	Abcam^b^
CD31	Mouse	1:500	ab9498	Abcam^b^
Mast cell tryptase	Rabbit	1:500	ab134932	Abcam^b^
**Secondary**				
Rabbit—AF555	Donkey	1:500	A32794	Invitrogen^a^
Rabbit—Cy5	Donkey	1:500	AP182S	Millipore^c^
Mouse—AF555	Donkey	1:500	A32773	Invitrogen^a^
Mouse—Cy5	Donkey	1:500	AP192S	Millipore^c^

CAMKII—CaM kinase II; VIP – Vasoactive Intestinal Peptide.

^a^Thermo Fisher Scientific, Rockford, Illinois, USA.

^b^Abcam, Cambridge, United Kingdom.

^c^Millipore, Burlington, Massachusetts, USA.

#### Microscopy and image analysis

Images were acquired employing either a laser-scanning microscope LSM 700 with ZEN Black SP1 2010B software (version 6.0.0.485) using 20×/0.8 Plan Apochromat, 40×/1.4 Plan Apochromat and 60×/1.46 αPlan Apochromat oil immersion objectives (Carl Zeiss, Jena, Germany) or a fluorescence microscope AxioImager Z1 equipped with 20×/0.5 Pol EC Plan-Neofluar objective and a digital camera AxioCamMRm running Zeiss AxioVision software (rel. 4.8.2; Carl Zeiss, Jena, Germany).

Image analysis was performed by freely available ImageJ software, version Java 1.8.0_172 (64 bit). Two-channel non-projected Z-stack images were used for the measurements. We did not count absolute numbers of neurons. Our goal was to identify the fraction of virus delivered eGFP expressing neurons labeled with a specific antibody. We are not dealing with a homogenously distributed population of cells since such eGFP expressing neurons are limited to the vicinity of the injection site. Therefore, we did not use an unbiased stereological approach. To obtain reliable and objective numbers of the antibody labelled fraction of eGFP expressing neurons, we used several methods. The number of neuronal somata exhibiting immunostaining was counted using the Cell Counter plug-in. In 6 parvalbumin antibody-stained sections we evaluated the density of virus delivered eGFP and secondary antibody fluorescence by taking the ratio of the background subtracted fluorescence to the manually measured somata cross-sectional area. Then, all somata with fluorescence density above the background level were considered as positive. These counts yielded the same result as simple manual count of visually identifiable neuronal somata. Thus, all counts were performed manually. In these counts, we did not rely solely on appearance of yellow pixels in the superimposed images, only neurons clearly and independently identifiable in each layer (eGFP and antibody) were counted. In addition, it was verified that the depth of antibody stain corresponded to the depth of the eGFP+ neuron. Two researchers made independent counts in most slices.

**Figure 2:**
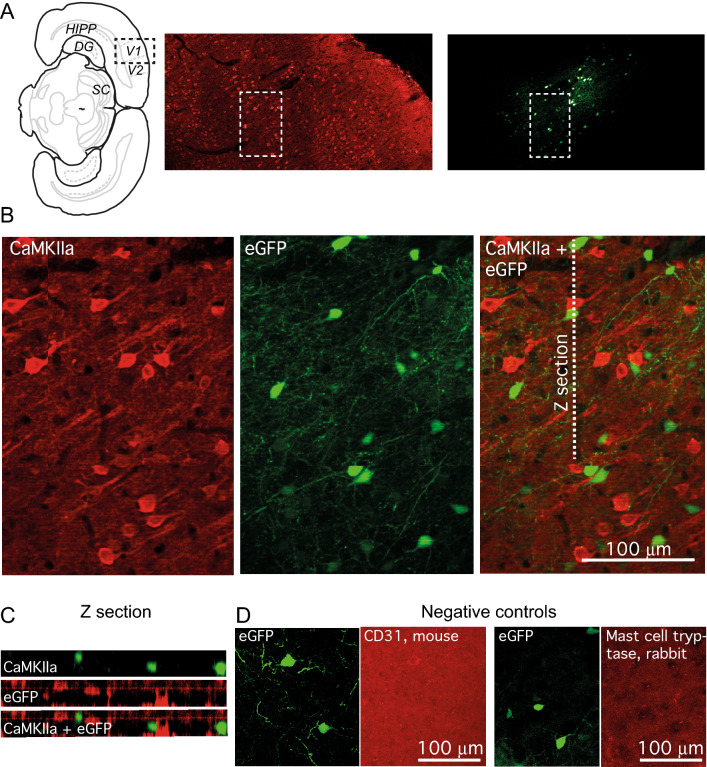
PV promoter excludes the reporter expression in pyramidal neurons. (**A**) Schematic representation of rat brain coronal section with delineated brain areas: V1, visual cortex area V1, HIPP, hippocampus, DG, dentate gyrus. The black dashed line box on the right indicates the approximate location of brain sections shown on the right for CamKIIa antibody (red, middle) and the virus delivered eGFP (green, right). White dashed line box indicate the area shown in (**B**) Fluorescence images of a brain section stained for CamKIIa antibody (left), the virus reporter fluorescence (middle) and merged two images (right). (**C**) A Z section along the dashed line shown in (**B**). The scale is the same as in (**B**). (**D**) Antibodies for CD31 protein (platelet endothelial cell adhesion molecule) were used as a negative control for mouse antibodies while mast cell tryptase antibodies were used as negative control for rabbit antibodies. Note the lack of any cell-specific staining.

**Figure 3:**
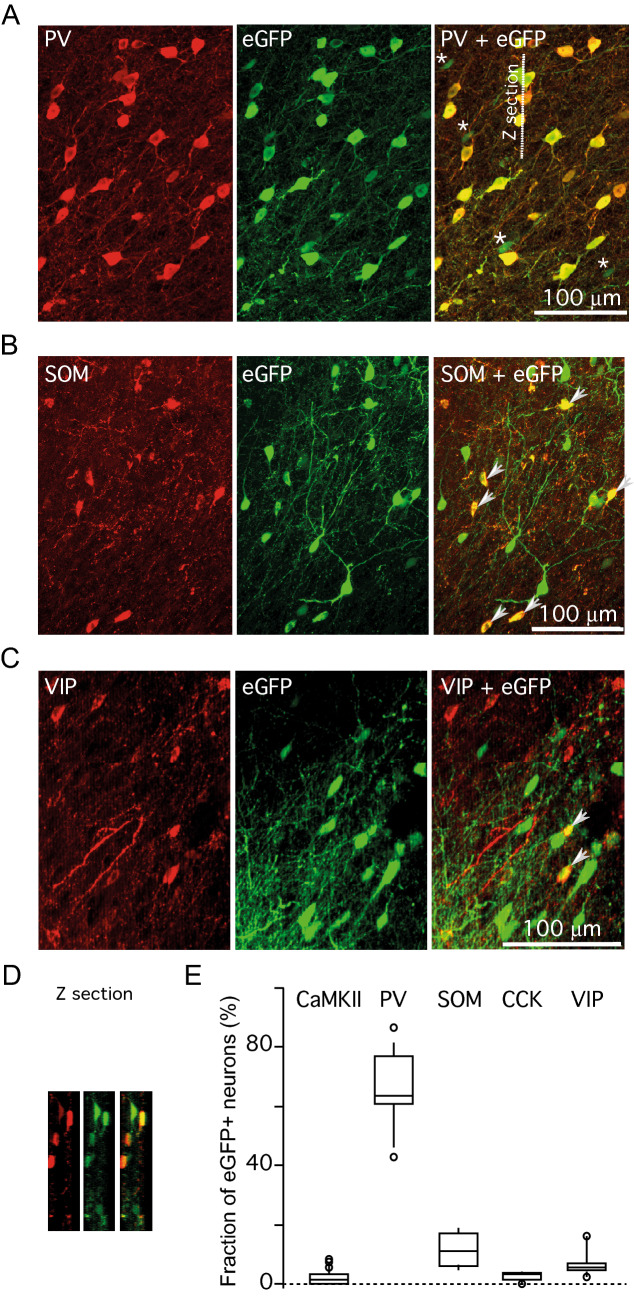
PV promoter driven eGFP is mainly expressed in PV positive neurons. (**A**) Fluorescence images of a brain section stained for PV antibody (left), the virus reporter eGFP fluorescence (middle) and merged two images (right). Most the rAAV eGFP containing neuronal somata were also positive for PV as indicated by the yellow merged colour. The few rAAV eGFP cells negative for PV are indicated by asterisks. (**B**, **C**) Fluorescence images of a brain section stained for SOM (in B) or VIP (in **C**) antibody (left), the virus reporter eGFP fluorescence (middle) and merged two images (right). Arrows indicate neuronal somata that contain both the rAAV eGFP and SOM or VIP antibody fluorescence. (**D**) A sample of a Z section along the dashed line shown in (**A**). (**E**) A box and whisker plot summarizing co-expression data. The whiskers indicate 10–90% range.

Microphotographs for the figures were generated by adjusting image size, brightness and contrast using Adobe Illustrator (Adobe Systems, San Jose, USA), the same adjustments were made prior quantification. For Z section of CaMKIIa antibody stain (Fig. 2C) we adjusted the fluorescence intensity of horizontal sections to compensate the limited penetration of antibodies: the fluorescence intensity of each layer was normalized to have the peak value of the pixel fluorescence intensity distribution the same across all layers. No such adjustment was made for Z section of the PV antibody stain (Fig. 3D). Brain structures were identified according to a stereotaxic atlas of the rat brain^28^.

### Optogenetic experiments

All procedures were carried out in accordance with the European Communities Council Directive of 22 September 2010 on the protection of animals used for scientific purposes (2010/63/EEC) and were approved by the Animal Care and Use Committee of the State Food and Veterinary Service of Lithuania (No. G2-147 of May 13, 2020). Wistar rats used in these experiments were injected with PV_CHR134_eYFP viral particles into V1 area as described above at least 3 weeks before the recording session. Standard procedures were used for single unit recordings^29,30^. Briefly, rats were anesthetised with urethane (Alfa Aesar—Thermo Fisher, Karlsruhe, Germany, 1.2–1.7 g/kg) aided with butorphanol (Richter Pharma AG, Wels, Austria, 0.4 g/kg), both delivered intraperitoneally^31^. The depth of anesthesia was monitored by testing for the absence of hind limb withdrawal reflex following a pinch of the paw. To maintain the depth of anesthesia, additional doses of butorphanol were used as needed for the duration of the experiment. Under anesthesia, the body temperature was maintained at 36–38 °C with a heating pad. The anesthetized animal was placed in a modified stereotaxic apparatus (World Precision Instruments, Sarasota, FL, USA) that enabled unobstructed view of the left eye. Eye gel was applied to avoid eye drying. Although eye movements are rarely a problem in anesthetized rats^32^, to prevent any eye movements and to maintain lids open, miniature hooks were inserted between the conjunctiva of the inner eyelids and the sclera and then attached to the stereotaxic frame with a thread. To dilate pupils, atropine (Sigma-Aldrich Chemie GmbH, Taufkirchen, Germany) solution of 0.5% was applied to the cornea surface.

For recording, a small craniotomy (approximately 2 × 2 mm) was made in the parietal bone and a small incision in the dura mater was made. Tetrodes from Thomas Recording (Giessen, Germany) were used to acquire action potential data while a blue PlexBright LED of 465 nm (Plexon, Dals, Texas, USA) was used to excite neurons expressing channelrhodopsins. The LED light was delivered via a 105 µm optic fibre (Thorlabs GmbH, Munich, Germany) glued to the tetrode in such a way that the fibre tip was located ~ 200 µm above the tetrode tip. Electrodes were typically placed 1.8 –2.8 mm rostral to lambda and 2.5–3.5 mm lateral to the midline, and then lowered perpendicular to the cortical surface by means of a micro-drive to a depth of > ~ 500 µm. For data acquisition, a 4-channel differential amplifier was used (EX4-400, DAGAN, Minneapolis, MN, USA) with a band-pass filters set to 300–10,000 Hz. Data were acquired via a National Instrument DAQ card PCI-MIO-16E-1 (6070E) connected to a PC at 40 kHz sampling frequency and visualized by employing a custom program written in the Labview environment (National Instruments, Austin, TX, USA). Although sampling rate of 40 kHz in theory is excessive, tests showed that it improves signal to noise ratio, presumably because the real signal has very high frequency components that are not completely cancelled out by the analogous filters of the amplifier.

For visual stimulation a LED backlit LCD monitor (frame rate 60 Hz, 58 cm × 28 cm) was used for image presentation and was placed 16 cm from the right eye. The bottom of the monitor was slightly (~ 5 cm) below the rat eye level. The monitor was inclined at 45° angle to the rat’s longitudinal axis in the horizontal plane. In the vertical plane the monitor was inclined at 30° towards the rat in order to cover a wider range of vertical angles. The full screen subtended 110 horizontally and about 80° vertically. At the center of the screen 1 cm corresponded to ~ 3.6o of visual angle. The monitor had 1920 × 1080 image pixels or > 30 pixels/cm corresponding to a minimal stimulus size of < 0.1°, well below visual acuity of ~ 0.5°–1° found in rodents^33^. Images were generated by employing an open-source software package PsychoPy, controlled by an in-house program written in Python and synchronized with recordings via the Labview environment (National Instruments, Austin, TX, USA)^34^.

All visual stimuli were bright images, ~ 30–45 cd/m^2^ at rat’s eye level, presented on a dark grey background (~ 0.3–0.45 cd/m^2^). The visual stimulation procedure started with the receptive field (RF) mapping that was performed by flashing 10° wide bright round spots for 600 ms followed by a 1900 ms gap (2.5 s inter-stimulus interval, ISI) on a 12 × 11 grid in a quasi-random fashion. Having determined RF, visual stimuli were presented inside RF. At least 5 repetitions were used for each test. Extracellular data analysis on recorded traces was performed with custom written routines by employing Igor Pro 6.3.7.2 software (Wavemetrics, Lake Oswego, Oregon, USA) as described previously^29^.

### Statistical analysis

The data are presented as absolute numbers (n), percentages and mean (M) ± standard error (SE). The Kruskal–Wallis test was employed when comparing data of multiple groups; differences between groups was compared by Mann_Whitney U test. A p-value of < 0.05 was used as a criterion for statistical significance, all p values below 0.0001 were reported as < 0.0001. The statistical package of Igor Pro 6.3.7.2 (Wavemetrics, Lake Oswego, Oregon, USA) was used for statistical analysis.

## Results

### Promoter design

To identify elements potentially regulating gene expression, we focused on the sequences conserved among several mammalian species. Genomic DNA coding for parvalbumin and upstream 15 kb region of Rattus norvegicus, Mus musculus and Homo sapience were aligned. We picked sequences with the high level of homology. There are 5 blocks of such sequences spanning about 6 kb of genomic DNA. We composed our promoter (size 1.97 kb) from these blocks of Rattus norvegicus genomic DNA and used it in rAAV construct expressing eGFP fluorescent protein (Fig. 1).

3′untranslated region of PV mRNA (size 226 bp) showed high homology also. We used this sequence instead of hGH poly(A).

### Immunohistochemical analysis of virus delivered eGFP expressing neurons

In this analysis we aimed to answer two questions. First, how well our promoter restricts gene expression to interneurons. Second, whether there is a strong preference for viral gene expression in one class of cortical interneurons. In the cortex all principal cells express calcium/calmodulin-dependent protein kinase type II (CaMKII) ^16,35^. We used a CaMKII alpha subunit antibody to answer the first question. As shown in Fig. 2, very few cells expressing the virus delivered eGFP were also expressing CaMKII alpha subunit. In fact, only 2.1 ± 0.5% of neurons expressing eGFP possessed a detectable level of CaMKIIa fluorescence (median = 1.4%, Q1 = 0%, Q3 = 3.1%, 3 rats, 23 slices, 32 out of 1336). Thus, > 97% of the virus delivered eGFP expressing neurons are non-pyramidal, presumably GABAergic neurons, because almost all interneurons are GABAergic in the cortex ^16,35,36^ and no expression of eGFP was observed in non-neuronal cells that differed morphologically from the neurons in agreement with previous studies on rAAV2 serotype selectivity ^37–40^.

Since the virus delivered eGFP expression was found almost exclusively in non-pyramidal cells, presumably in interneurons, next we tried to determine in which interneuron classes the eGFP expression is prevalent. It has been established that almost all cortical interneurons can be assigned to three classes, each expressing a specific marker. Approximately one third of cortical interneurons express a Ca^2+^ binding protein parvalbumin (PV), another third express neuropeptide somatostatin (SOM) and the rest of interneurons express the ionotropic serotonin receptor 5HT3a (5HT3aR) ^36^. There are well established antibodies for all these markers except for 5HT3a receptor; we did not find suitable antibodies for 5HT3aR. However, 5HT3aR expressing interneurons also co-express several other markers such as vasoactive intestinal peptide (VIP), cholecystokinin (CCK), reelin, a calcium binding protein calretinin (CR) and neuropeptide (NPY). CCK and VIP are expressed almost exclusively in 5HT3aR neurons ^36,41^. The other markers are less restricted to 5HT3aR neurons although in rats CR is possibly expressed mostly in these cells; however, direct evidence is lacking ^41,42^. Therefore, we used CCK and VIP as a proxy for an estimate of eGFP expression in the 5HT3aR group of neurons.

Figure 3 shows examples of these tests, while Table 2 provides the summary of data. As expected, most eGFP fluorescent neurons were also expressing PV: 66.5 ± 2.8% (median = 65.3%, Q1 = 59.5%, Q3 = 76.2%, 6 rats, 17 slices, 977 out of 1440). We performed both the visual counts and the density counts of PV antibody fluorescence, both measures yielded similar results. A substantial fraction of eGFP neurons were also positive for SOM antibody: 14.6 ± 2.4% (median = 65.3%, Q1 = 59.5%, Q3 = 76.2%, 7 rats, 21 slices, 202 out of 1486). The fraction of VIP expressing neurons among eGFP fluorescent cells was significantly smaller: 7.1 ± 1.2% (median = 6.6%, Q1 = 4.5%, Q3 = 8.8%, 6 rats, 10 slices, 46 out of 737). The incidence of CCK antibody labelled neurons among the virus delivered eGFP expressing cells was even lower, 2.8 ± 0.6% (median = 3.1%, Q1 = 1.3%, Q3 = 3.6%, 5 rats, 11 slices, 23 out of 809). The overall differences between the incidences of the tested neuronal markers among the virus delivered eGFP expressing neurons were highly significant, the Kruskal Wallis H test between groups: Hdf = 61.2, p < 1e − 11. There was no significant difference between the incidences of CCK and CaMKIIa, SOM and VIP and CCK and VIP positive neurons among eGFP labelled cells (p > 0.05). The corresponding p values for all pairs of groups are presented in Table 3.

**Table 2:** Summary of rAAV2 PV-eGFP co-expression in area V1/V2.

Marker	Number of rats	Number of slices	Total number of eGFP fluorescent neurons	Number of eGFP neurons labelled with antibody	Fraction of antibody labeled neurons
CAMKIIa	3	23	1336	32	2.1 ± 0.5%
Parvalbumin	5	16	1410	957	66.5 ± 3.0%
Somatostatin	6	18	1404	171	11.8 ± 1.3%
VIP	5	8	618	35	6.6 ± 1.4%
Cholecystokinin	4	8	590	16	2.6 ± 0.5%

**Table 3:** Statistical significance between fractions of antibody labeled neurons (p values).

Marker	CamKIIa	Parvalbumin	Somatostatin	VIP	Cholecystokinin
CAMKIIa	1	< 0.0001	< 0.0001	< 0.004	< 0.25
Parvalbumin	< 0.0001	1	< 0.0001	< 0.0001	< 0.0001
Somatostatin	< 0.0001	< 0.0001	1	< 0.2	< 0.0001
VIP	< 0.004	< 0.0001	< 0.2	1	< 0.005
Cholecystokinin	1	< 0.0001	< 0.0001	< 0.005	1

The observed specificity of the virus-delivered protein expression was observed also in other cortical area (Table 4) and in the hippocampus (Table 5). In the hippocampus the major interneuron groups are positive for either parvalbumin, somatostatin or nNOS (neuronal nitric oxide synthase)^43^. Interestingly, we found no co-localization of the virus delivered eGFP and nNOS while there was a higher fraction of SOM positive neurons among eGFP fluorescent cells in the hippocampus compared to the cortex (35.2 ± 3.2% and 11.9 ± 1.3% respectively, p < 0.0001, Table 5). Meanwhile, the fraction of PV positive cells was almost identical, 66.5 ± 3.0% and 68.0 ± 1.9% respectively (p > 0.5).

**Table 4:** Summary of rAAV2-PV -eGFP co-expression in area S1.

Marker	Number of rats	Number of slices	Total number of eYFP fluorescent neurons	Number of eGFP neurons labelled with antibody	Fraction of antibody labeled neurons
CAMKIIa	1	6	146	0	0.0 ± 0.0%
Parvalbumin	2	9	427	273	69.9 ± 5.0%

**Table 5:** Summary of rAAV2-PV-eGFP co-expression in hippocampus.

Marker	Number of rats	Number of slices	Total number of eYFP fluorescent neurons	Number of eGFP neurons labelled with antibody	Fraction of antibody labeled neurons
CAMKIIa	3	12	375	7	2.2 ± 1.5%
Parvalbumin	3	22	1099	744	68.0 ± 1.9%
Somatostatin	3	12	491	166	33.3 ± 2.8%
nNOS	2	6	77	0	0.0 ± 0.0%

### Optogenetic tests

**Figure 4:**
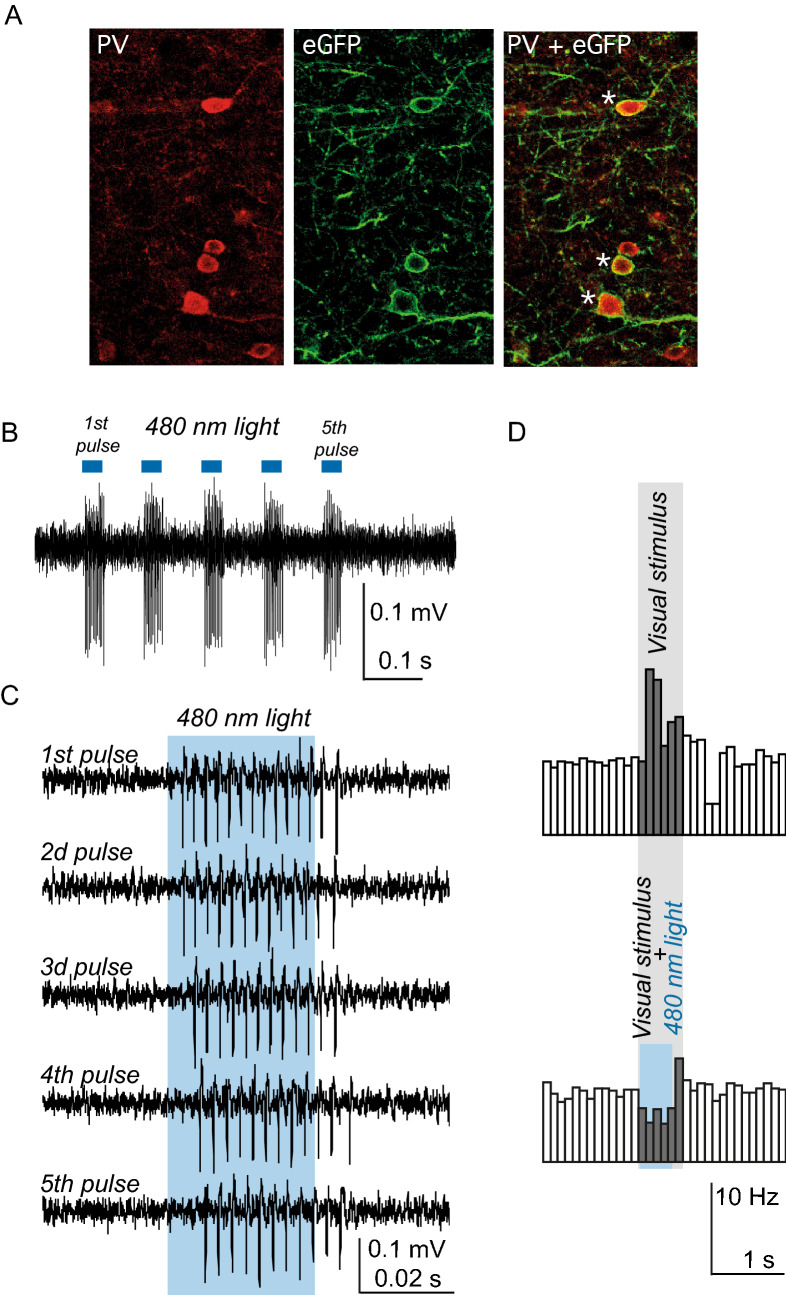
rAAV5-PV-ChR2-eYFP construct driven gene expression was mainly limited to PV positive cells and was sufficient for optogenetic tests. (**A**) Fluorescence images of a brain section stained for PV antibody (left), the virus reporter eYFP fluorescence (middle) and merged two images (right). A single section of a confocal image is shown. Asterisks indicate V positive neurons that were also expressing ChR2-eYFP. Note, that the fluorescent probe is largely membrane bound because it is covalently attached to the channel. (**B**, **C**) PV neurons expressing mutant ChR2 (H134R mutation) were activated by trains of 480 nm LED light. In (**B**) a trace of extracellular recording for putative fast spiking PV neurons is shown. In (**C**) the same trace is shown on expanded time scale to discern single action potentials generated by a putative PV neuron during light stimulation. (**C**) In a separate experiment, principal neurons responded to light stimulation. A peristimulus histogram (PSTH) is shown. In the upper PSTH only a visual stimulus was presented during the time indicated by a grey shade. Meanwhile, in the lower PSTH a presentation of visual stimulus was combined with the 480 nm LED stimulation shown in light blue shade. Note the disappearance of visual response in these putative principal neurons (dark bars of the PSTH).

Optogenetics is one of the main intended uses of our construct. Therefore, we tested the ability of our construct to drive sufficient levels of expression of light sensitive channels that are used in such experiments. To this end, in our rAAV construct we added a modified channelrhodopsin channel ChR2 with a point mutation H134R and a covalently attached eYFP probe^23^. In addition, to test whether an rAAV serotype had any effect on the selectivity of the virus driven gene expression, we chose rAAV5 instead of rAAV2 used in previous tests. Immunohistochemical analysis confirmed that the expression of the ChR2 channel-attached eYFP was excluded from CaMKII positive cells and was mainly found in PV positive neurons (Fig. 4A and Table 6). Optogenetic tests showed that the levels of ChR2 channel expression were sufficient to achieve high frequency responses of the putative fast spiking PV neurons (Fig. 4B,C) while the activation of interneurons led to a complete elimination of visual responses in area V1 principal neurons (Fig. 4D).

**Table 6:** Summary of rAAV5-PV-ChR2H134R-eYFP co-expression in area V1/V2.

Marker	Number of rats	Number of slices	Total number of eYFP fluorescent neurons	Number of eGFP neurons labelled with antibody	Fraction of antibody labeled neurons
CAMKIIa	3	17	710	0	0.0 ± 0.0%
Parvalbumin	3	20	936	637	69.7 ± 3.0%

## Discussion

We designed an rAAV construct for a highly specific rAAV-delivered gene expression in cortical interneurons: less than 3% of the virus delivered eGFP fluorescence was found in cortical principal cells, pyramidal neurons, suggesting that > 95% of the virus delivered gene expression occurred in cortical interneurons. Moreover, ~ 2/3 of the virus delivered eGFP fluorescence was detected in PV expressing interneurons. This pattern of expression of the rAAV-delivered genes persisted after a change in the rAAV serotype and gene, indicating that the combination of the PV promoter and PV poly(A) sequence was responsible for the expression specificity. The remaining ~ 1/3 of the neurons expressing AAV-delivered eGFP seems to be evenly distributed between two other major groups of cortical interneurons, SOM expressing cells (~ 15%) and 5HT3aR expressing neurons. We do not have direct data on the incidence of 5HT3aR expressing neurons among the virus delivered eGFP expressing cells because of the lack of good quality antibodies for 5HT3aR ^35^. However, > ~ 40% of 5HT3aR expressing neurons are positive forVIP ^36,41^, while a much smaller fraction, < 20%, are also positive for CCK^35,41^. Since there were approximately 7% of VIP positive and ~ 3% of CCK positive neurons among the virus delivered eGFP expressing cells, it is likely that ~ 15% of the eGFP fluorescent neurons were also expressing 5HT3aR. This suggestion agrees with the notion, that the total number of all types of eGFP expressing cells should be 100%: 67% PV + 3% CaMKII + 15% SOM + 15% 5HT3a = 100%. Similar pattern of exclusion of principal neurons and strong preference for PV interneurons was also found also in the cortical area S1 (Table 4) and hippocampus (Table 5) suggesting that the achieved selectivity of the rAAV construct is not limited to the cortical area V1/V2. Nevertheless, additional tests in other brain regions and other species are required for more broad use of this construct.

Two recent papers have described promoters with higher specificity for PV neurons ^14,20^. Nevertheless, we achieved slightly higher exclusion of principal neurons, at least in cortex: the E2 promoter of Schneider et al. has > 90% specificity for GABA interneurons and the mDlx enhancer selectivity was between 90 and 92% in several animal species and 98% in juvenile ferrets ^14^, in our case this number is most likely to be above 95%. We base this assumption on CaMKIIa data (2.8% overlap) and the fact that only < 1% of cortical neurons are neither pyramidal cells nor GABAergic interneurons ^35,36^. The second paper used a more complex strategy to achieve 70% specificity of h56D promoter in cortical PV neurons: only when promoters from two AAV viruses were active in a cell the expression of the target protein occurred ^20^. Interestingly, that for one of these two viruses a similar strategy was used: the authors searched for conserved regions downstream to PaqR4 gene that is highly specific to PV neurons. In this case, however, this strategy alone did not yield a promoter that could restrict gene expression to PV neurons. Therefore, the authors resorted to the combinatorial approach of two viruses. Thus, knowledge about specificity of such sequences in driving gene expression, including ours, may help to design even more specific promoters, both for research and clinical application. Finally, these data can help to determine the mechanisms of promoter function.

## Conclusion

We demonstrate that the designed construct is capable of highly selective expression of rAAV proteins in cortical interneurons with a strong preference for PV positive cells.

## Supplementary Information

Supplementary Information.

## Data Availability

The author declares that all data necessary for evaluation of the statements of this paper are present in the manuscript. However, any additional reasonable requests for raw data files will be granted by the corresponding author.
