# Supplementary material for: An efficient rAAV vector for protein expression in cortical parvalbumin expressing interneurons
Source: Sci Rep. 2022 Oct 25;12:17851. doi: 10.1038/s41598-022-21867-0 (PMC9596399; doi:10.1038/s41598-022-21867-0)
Supplement: Supplementary file 1 — Supplementary Information. [file 41598_2022_21867_MOESM1_ESM.pdf]

Supplementary information

Title:

An efficient rAAV vector for protein expression in cortical parvalbumin expressing interneurons

Tatiana Tkatch, Kristina Rysevaite-Kyguoliene, Ignas Sabeckis, Deimante Sabeckiene, Dainius H Pauza, Gytis Baranauskas

PV promoter full sequence (1.97 kbp):

acggggagaatcagctcaggatctgcctgctgggaatggggcagagattccttgagttaatgtacgtcacccca
cacgactaacctcataaatatcactggatctgcaggtccacctgcggccagcgccctggaccgcagcagctct
gtgtgtatcctgttccctgccgcagcaggtggaaatagcgtgaaccaaggaaatctcaccagcaaagataaca
gctgaacgcttgtctcctggcagacagtggctaagagaatgctgacccacacgcacaccacacacatgggtcc
ctgccctctccagggtctggaaggcagagtgggctttggacataagtgcatatggttgaccaggctgggtgaac
ctggacaagtgacttggcttctctgaacctcagttgtgctcatctgtggagaagattcatttggggaatgtatgtatga
gtgtacaagactgactaggttaatcattcaagactgccaggattaacacggacttgaagtggaaggactggaa
aatattttaggactcactacttactttctaactatgtgaccttggacaaagttctaaacatctctgagattctatctcttca
aatttctgaggtctttgtaagggcacagtggagtgatacgcctagatcacaagagcagtaggtacccccacccc
acagtgtctcagggtacagtaaagacacagtatgtgtaggggacctgacctagtccctttaccccaagcaacct
ctgttcctttccttccctcttcctttccttgcaagtttagaacaaagtgtggctgccacccaaggtgatcatggaaggct
tcatgcaagaggtgtgccctgcttggaccttattgctgggtgctgtagggaggtagaggccaagggttattaatag
gcctggctcaataggcctggatgggggtgaatgtgatacagacacccagcaccgtggctggggagtacctgac
accggaaggggagggggccaggggctgggggagcgccacctcccaaaatagccaaggctgtagactattt
aagtgactgtcccaccacaagtctcatttcccaacaggatctcccaccagcccagcttttctatataggctctgacc
tctggtcatccaaggtaagtggtagtctctgaggttagaggatggagaaggcagaatcaggccaattgggagg
gatagccatgccgctatttctctagagactccaggctcactatggcacgagggtctccaggtggagaaacagat
gctgtaatgaggctacagggtagagagagctggacattgagctatcagcacagaagcctggctcattggtactc
gcagtccagctgggaacaagggacagaggctgaggacactgagtcaactgagataggagccataggacag
ctagggcagctgaagggtccaggacagctgggggggtctggagtgggcctcatgcccagagctatgaagtcag
gtcttagggccttggaggccagctggcaggctctggcccgaagaagctgactcaccaggagggggcagctgg
agccaggctctcctaaggtttccaggaagggcagccttccagggctcagtcatgggagacagtgttgacagca
agttgtcaggcaacttgagctactgggcagctgggaagttgtcccttggttcccaataccatcaccatcccagatg
ctgcccacctgcctcaggtcccacacaggatcctgatcaagtcccctgaatgcctaggaacccatgggcgtgtg
acccgttcagcttgaaggtgtcgccgggactgagctgctgagctgtgtgcggagctccgcgccccctggcgcgg
agccaggccgggcgggcgtgcgcctggcgcgcgacactgcagcgctggtcatatgagcagaaaggatgag
aagaacactttttaatcttttcgcacttgctctgcccgcttaa

PV poly(A) full sequence (226 bp):

tggccgaaagctaagtggcgctgactgcttgggtctcccacctctccacccccccatgccccatctcagcccttctc
gcggccctcctgggtttctgttcagtttgtttatgttattttttactcccccatcctttatggccctcgaatgacaccactctt
ctggaaaatgctggagaaacaataaaggctgtacccatcggacaccacctgtagggaggacccagg

Rat age, weight and sex used for injections:

Virus, injection site	Antibody	Rat number	Sex	Age (months)	Weight (g)
PV_eGFP, V1/V2	Parvalbumin	1.	Male	2.0	229
		2.	Male	2.1	180
		3.	Male	2.5	228
		4.	Male	2.5	337
		5.	Male	2.8	342
	Somatostatin	1.	Male	1.9	217
		2.	Male	1.9	180
		3.	Male	2.1	180
		4.	Male	2.5	337
		5.	Male	2.8	342
		6.	Male	3.3	359
	CaMKIIa	1.	Female	2.3	195
		2.	Female	2.5	192
		3.	Male	2.7	390
	CCK	1.	Male	2.0	229
		2.	Male	2.5	337
		3.	Male	2.8	342
		4.	Male	3.3	359
	VIP	1.	Male	1.9	229
		2.	Male	1.9	180
		3.	Male	2.5	337
		4.	Male	2.8	342
		5.	Male	3.3	359
PV_eGFP, Hippocampus	Parvalbumin	1.	Female	2.3	199
		2.	Female	3.1	272
		3.	Female	3.8	288
	CaMKIIa	1.	Female	3.1	272
		2.	Female	3.8	288
		3.	Female	2.9	265
	SOM	1.	Female	3.8	288
		2.	Female	2.3	199
		3.	Female	2.9	265
	nNOS	1.	Female	3.1	272
		2.	Female	3.8	288
PV_CHR134, V1/V2	Parvalbumin	1.	Female	2.0	180
		2.	Female	3.0	180
		3.	Female	4.0	197
	CaMKIIa	1.	Female	2.0	180
		2.	Female	3.0	180
		3.	Female	4.0	197
PV_eGFP, S1 cortex	Parvalbumin	1.	Female	2.9	265
		2.	Female	3.1	272
	CaMKIIa	1.	Female	2.9	265
